# Spatio-Temporal Distribution of Smads and Role of Smads/TGF-β/BMP-4 in the Regulation of Mouse Bladder Organogenesis

**DOI:** 10.1371/journal.pone.0061340

**Published:** 2013-04-19

**Authors:** Syed S. Islam, Reza Bayat Mokhtari, Sushil Kumar, Joe Maalouf, Sara Arab, Herman Yeger, Walid A. Farhat

**Affiliations:** 1 Developmental and Stem Cell Biology, The Hospital for Sick Children Research Institute, Toronto, ON, Canada; 2 Department of Pediatric Laboratory Medicine, The Hospital for Sick Children Research Institute, Toronto, ON, Canada; 3 Physiology and Experimental Medicine, The Hospital for Sick Children Research Institute, Toronto, ON, Canada; 4 University of Toronto, Toronto, ON, Canada; Simon Fraser University, Canada

## Abstract

Although Shh, TGF-β and BMP-4 regulate radial patterning of the bladder mesenchyme and smooth muscle differentiation, it is not known what transcription factors, local environmental cues or signaling cascades mediate bladder smooth muscle differentiation. We investigated the expression patterns of signaling mediated by Smad2 and Smad3 in the mouse embryonic bladder from E12.5 to E16.5 by using qRT-PCR, *in situ* hybridization and antibodies specifically recognizing individual Smad proteins. The role of Smad2 and Smad3 during smooth muscle formation was examined by disrupting the Smad2/3 signaling pathway using TβR1 inhibitor SB-431542 in organ culture system. qRT-PCR results showed that R-Smads, Co-Smad and I-Smads were all expressed during bladder development. RNA *ISH* for BMP-4 and immunostaining of TGF-β1 showed that BMP-4 and TGF-β1 were expressed in the transitional epithelium, lamina propia and muscularis mucosa. Smad1, Smad5 and Smad8 were first expressed in the bladder epithelium and continued to be expressed in the transitional epithelium, muscularis mesenchyme and lamina propia as the bladder developed. Smad2, Smad3 and Smad4 were first detected in the bladder epithelium and subsequently were expressed in the muscularis mesenchyme and lamina propia. Smad6 and Smad7 showed overlapping expression with R-Smads, which are critical for bladder development. In bladder explants (E12.5 to E16.5) culture, Smad2 and Smad3 were found localized within the nuclei, suggesting critical transcriptional regulatory effects during bladder development. E12.5 to E16.5 bladders were cultured with and without TβR1 inhibitor SB-431542 and assessed by qRT-PCR and immunofluorescence. After three days in culture in SB-431542, α-SMA, Smad2 and Smad3 expressions were significantly decreased compared with controls, however, with no significant changes in the expression of smooth muscle myosin heavy chain (SM-Myh. Based on the Smad expression patterns, we suggest that individual or combinations of Smads may be necessary during mouse bladder organogenesis and may be critical mediators for bladder smooth muscle differentiation.

## Introduction

**Figure 1:**
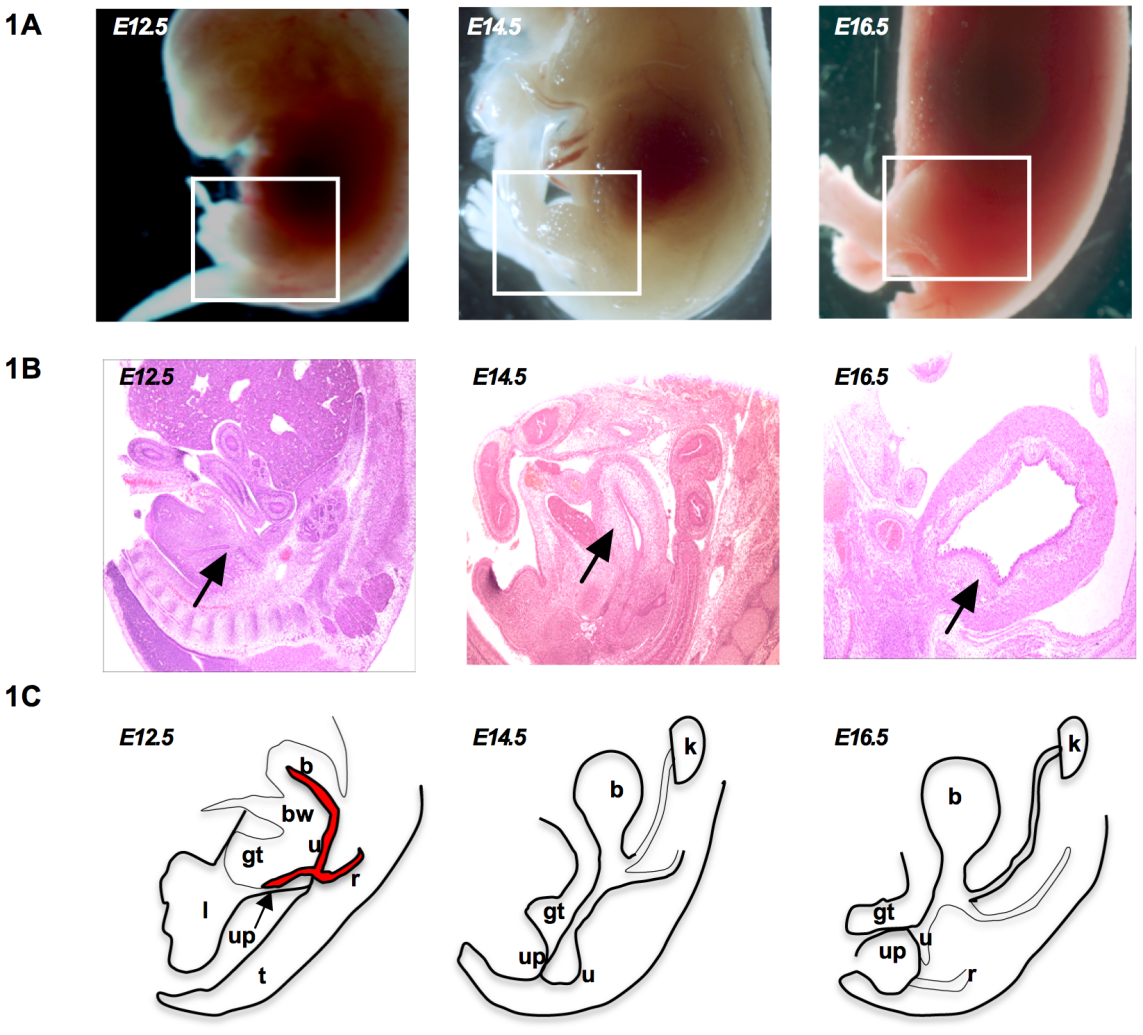
Schematic of depicting urogenital organs adjacent to the limb and the tail at E12.5 to E16.5. **A**) Whole embryos from E12.5 to E16.5 **B**) H & E sections from E12.5 to E16.5 and **C**) Bladder developmental progression. b: bladder, bw: body wall, u: urethra, gt: genital tubercle, up: urethral plate, r: rectuml, limb, t: tail, k: kidney.

The bladder is a complex organ that develops from the caudal part of the hindgut and first appears at about embryonic day 9.5 (E9.5) of mouse development. At E10.5, the whole region dilates to form the cloaca and initially possesses an endodermal lining. At E10.5, the urorectal septum is visible and it subdivides the cloaca into the urogenital sinus (UGS) ventrally and the rectum as well as the anal canal dorsally. Figure 1 shows schematics of bladder development from E12.5 to E16.5. Around E13.5 to E14.5, the urogenital sinus epithelium differentiates into urothelium while the surrounding mesenchymal cells differentiate into smooth muscle cells [1], [2]. It has been established that the bladder epithelium greatly influences patterning of the bladder and that an epithelial signal is essential for induction of smooth muscle differentiation from bladder mesenchyme [3]. During bladder development, the undifferentiated mesenchyme differentiates into bladder smooth muscle cells [4]. It has been previously shown that urothelial and smooth muscle cells undergo differentiation in an orderly fashion defined by smooth muscle and Cytokeratin markers [3]. Given the orderly differentiation of the bladder layers, the mesenchymal-epithelial interactions likely play a role in the development of the epithelium, lamina propia and smooth muscle. But the mechanism by which the epithelium signals the mesenchyme in bladder development is not fully understood. It has been determined that TGF-β plays a critical role during bladder development. Transforming growth factor-β (TGF-β) have been shown to regulate cell growth and differentiation in both urothelium and bladder smooth muscle [5]. Studies have shown that TGF-β induced hyperplasia, upregulated collagen, inhibited proliferation of bladder smooth muscle cells [6] and modulated cellular phenotype in fibrosis. It has been shown to regulate connective tissue growth factor (CTGF) in bladder fibrosis [7].

TGF-β superfamily members engage in a wide range of critical biological activities, including cell proliferation, differentiation, motility, lineage determination and apoptosis. Members of the TGF-β family include TGF-β's, Nodal/Activin and bone morphogenetic proteins (BMPs) and signals through two heterodimeric complexes: Type I (TβRI) and Type II (TβRII) transmembrane serine-threonine protein kinase receptors. Signaling by TGF-β members is initiated by binding of ligands to specific receptors on the surface of the cell. Constitutively activated TβRII receptors activate TβRI kinase activity by phosphorylating TβRI and lead to activation of downstream intracellular signals of phosphorylated Smads transcription factors [8]. TGF-β and BMPs function through cytoplasmic signal transducer Smads, and different Smads play distinct roles in various cellular processes, including cell proliferation, differentiation and apoptosis [9]. Eight Smad family members are known in mammals and are classified as receptor-regulated Smads (R-Smads), common-group Smad (co-Smad) and inhibitory Smads (I-Smads), depending on their functionality. One group of R-Smads (Smad1, Smad5 and Smad8) transduces signals from the BMP signaling pathway, whereas another group of R-Smads (Smad2 and Smad3) mediates signals from TGF-β, activin and nodal signaling pathways. After phosphorylation by activated TGF-β receptor (TβRI), Smad2 and Smad3 form a complex with Smad4 and then shuttle to the nucleus to regulate transcription. It is thought that Smad3 contributes to most Smad-dependent responses to TGF-β in the adult, while Smad2 is critical during embryogenesis [9], [10]. The I-Smads (Smad6 and Smad7) are antagonists of TGF-β signaling, and the expression of Smad6 and Smad7 provides the negative feedback, which prevents formation of a complex with Smad4 and R-Smads [11], [12], [13], [14]. In order to understand the mechanisms of individual signaling pathways involved in biological processes small, inhibitory molecules are routinely used [15]. Recently, a potent TGF-β inhibitor SB-431542 has been shown to inhibit the *in vitro* phosphorylation of Smad2 and Smad3 [16] with no apparent direct effects on BMP signaling and/or ERK, JNK or p38 MAP kinase signaling.

Bladder smooth muscle cells form first in the outer zone of the peripheral mesenchyme, while the sub-epithelial mesenchymal region remains devoid of smooth muscle cells. Sonic hedgehog (Shh), BMP-4 and TGF-β are involved in smooth muscle differentiation and patterning in the bladder [17], [18]. The expression of *Gli* transcription factors that mediate Shh signaling, *Gli1* and *Gli2*, are temporally regulated in the developing bladder. For instance, *Gli1* is highly expressed in the sub-epithelial mesenchyme from E12.5 to E13.5, while *Gli2* expression in the peripheral mesenchyme increases from E12.5 to E15.5, but never reaches the level of expression seen in the sub-epithelial layer. The expression profile of BMP-4 in the peripheral mesenchyme follows the pattern of *Gli1*, but is delayed by one day [17]. TGF-β is highly expressed in the peripheral mesenchyme of the developing bladder, where smooth muscle cells differentiation occurs at E13.5 [17]. These findings support the hypothesis that high levels of BMP-4 may modulate smooth muscle formation in the sub-epithelial layer and differentiation in the adventitial region. High levels of Shh promote the proliferation of smooth muscle cells via *Gli2* in the inner mesenchymal region where BMP-4 inhibits these cells from complete differentiation. On the other hand, in the outer mesenchymal zone, where Shh signaling is low, exemplified by reduced expression of *Gli2* and BMP-4, smooth muscle cells differentiation occurs [18], suggesting that both BMP-4 and Shh show spatio-temporal activity in bladder development.

Despite current knowledge on the critical role of Shh, TGF-β and BMP signaling pathways in bladder development, little is known about Smad expression and downstream signal mediators, functions and the effects of SB-431542 for Smad inhibition during bladder development. Here, we show the spatial and temporal expression of various Smad transcription factors during bladder development by using bladder organ culture, qRT-PCR, *in situ* hybridization and immunostaining. We show that Smad expression in the mouse bladder starts at E12.5 and extends to E18.5, and that expression is continued until the bladder is completely formed. Smads are mostly expressed in the epithelium, lamina propria and muscularis mesenchymal cells. We also demonstrate that TβRI inhibitor SB-431542 significantly inhibits the TGF-β1 induced smooth muscle formation and downregulates phophorylated Smad2 and Smad3, which is essential for bladder smooth muscle formation during mouse bladder development.

## Results

### Temporal expression pattern of TGF-β1, BMP-4 and Smads in developing bladder

**Figure 2:**
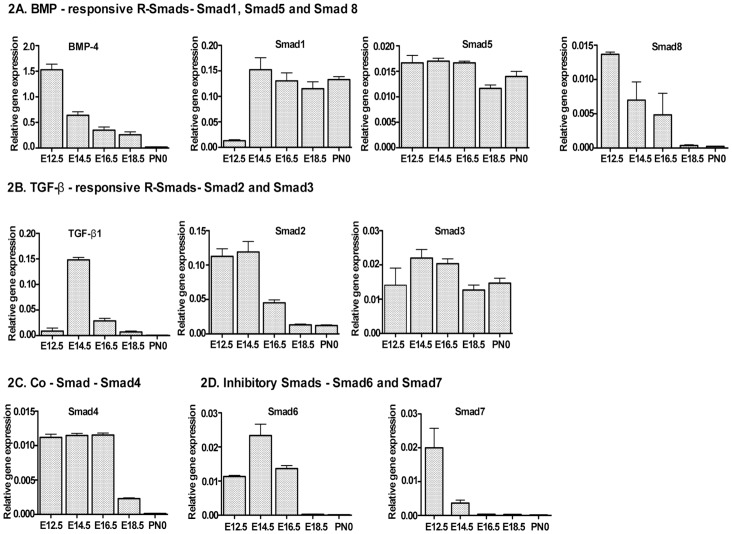
qRT-PCR analysis of the relative expression levels of TGF-β1, BMP-4 and Smads in embryonic bladders from E12.5 to neonatal day0. Ten embryonic bladders per group were lysed to extract total RNA for qRT-PCR. Data were normalized to that of GAPDH and the relative expression levels are shown. Each experiment was repeated three times and the mean value was calculated as expression value for each stage of embryonic bladder development. **A**) BMP-responsive R-Smads. Smad1, Smad5 and Smad8 were expressed in all gestation stages. Smad1 expression was minimal in E12.5 and Smad8 expression was low in E18.5 and neonatal day0 *(Mann-Whitney test, p<0.005)* **B**) TGF-β responsive R-Smads. Smad2 and Smad3 showed relatively high expression in all gestation stages while Smad2 showed decreased expression at E18.5 and neonatal day0. *(Mann-Whitney test, p<0.005)* **C**) Common Smad. Smad4 showed the highest expression pattern from E12.5 to E16.5 and decreased in E18.5 and neonatal day0 *(Mann-Whitney test, p<0.005)* and **D**) Inhibitory Smads. Smad6 and Smad7. Smad6 showed higher expression from E12.5 to E16.5, and lowest expression levels in E18.5 and neonatal day0 *(Mann-Whitney test, p<0.005)*, while Smad7 showed the highest expression level at E12.5 and decreased in the subsequent stages *(Mann-Whitney test, p<0.005)*.

BMP-4 and TGF-β1 are important regulators of urothelial proliferation and differentiation [19]. To investigate the role of Smad transcription factors in these processes, we determined their temporal expressions during bladder development. We first quantified the levels of BMP-4, TGF-β1 and Smads mRNAs by qRT-PCR analysis of embryonic day 12.5 to 18.5 (E12.5 to E18.5) and neonatal day 0 (PN0) total RNA. As for BMP-4 mRNA, the expression was high during early development of the bladder (E12.5/E14.5) and decreased significantly from E16.5 onward (Fig. 2A). BMP responsive Smad1 and Smad5 showed identical patterns of expression at E12.5, E14.5 and E16.5, except that Smad1 expression was very low at E12.5. Compared to Smad1 and Smad5, Smad8 expression was high at E12.5, started to decline from E14.5 and became undetectable at E18.5 and PN0 (Fig. 2A). TGF-β1 mRNA expression was low at E12.5 with a surge at E14.5 and rapid decline after E16.5 (Fig. 2B). In contrast, TGF-β responsive Smad2 expression was highest at E12.5 and E14.5, but fell significantly at later stages (Fig. 2B). By comparison, Smad3 showed a relatively constant expression through all stages with a peak at E14.5 and E16.5 (Fig. 2B). Co-Smad, Smad4 mRNA remained elevated from E12.5 to E16.5, but then declined significantly at E18.5 and became undetectable at PN0 (Fig. 2C). Inhibitory Smad6 expression was barely detectable at E18.5 and PN0 despite appreciable expression at earlier stages (Fig. 2D), while Smad7 peaked at E12.5 and declined significantly from E14.5 onward (Fig. 2D). These results illustrate a selective high expression of BMP-4 and TGF-β responsive Smads paralleling the absence of inhibitory Smads.

### Spatial expression pattern of BMP-4 and BMP responsive R-Smads, Smad1, Smad5 and Smad8 during bladder development

**Figure 3:**
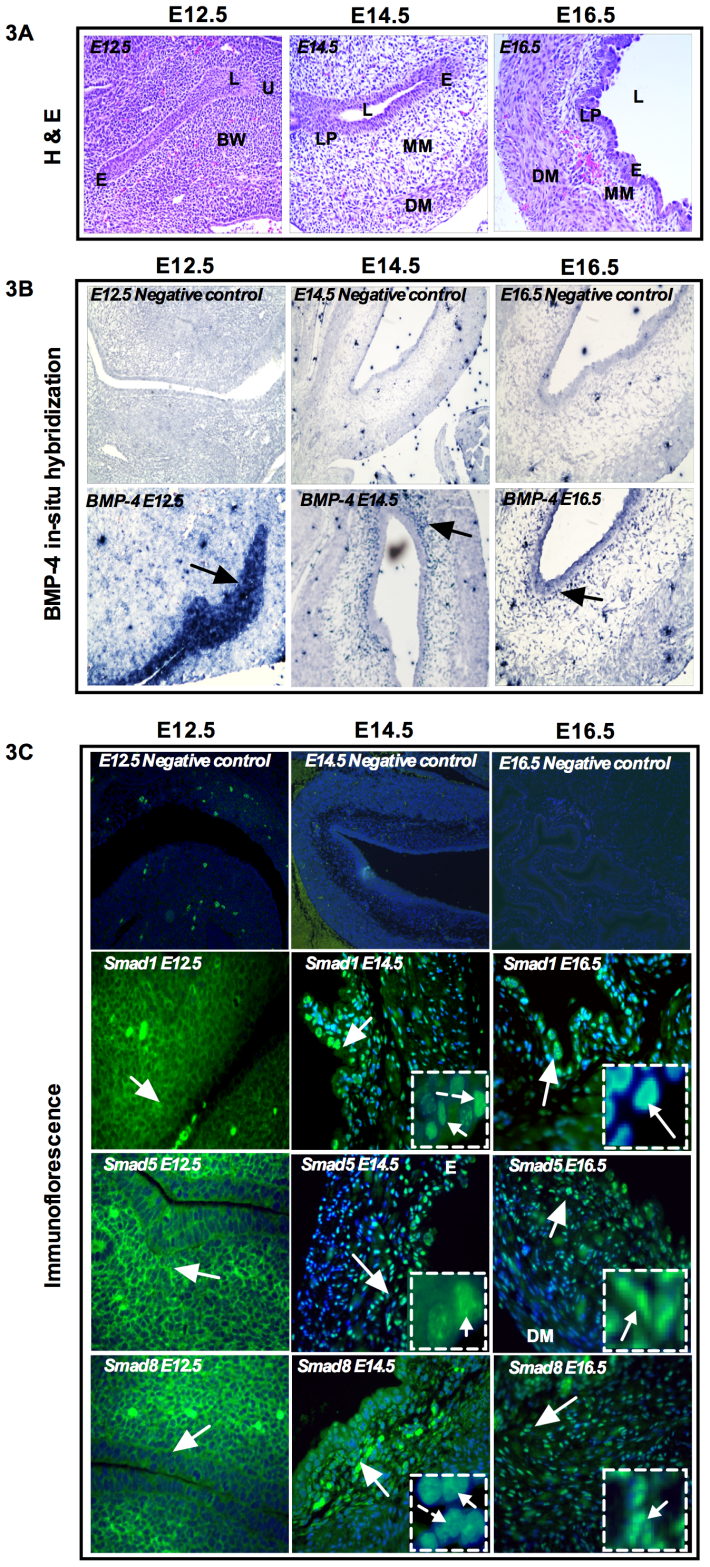
Expression pattern of BMP-4 and BMP-responsive Smads, Smad1, Smad5 and Smad8. **A**) Top panel shows H & E staining of mouse bladder anatomy from E12.5 to E16.5. **B**) *In situ* hybridization of BMP-4 (middle panel). Negative controls of BMP-4 (top middle panel). No BMP-4 expression was detected for negative control from E12.5 to E16.5. At E12.5, BMP-4 was localized in the bladder epithelium and urethra (arrow). At E14.5, BMP-4 was localized adjacent to the submucosa (lamina propia) and muscularis mesenchyme and periphery of the detrusor muscle (arrow). At E16.5 BMP-4 expression was similar to E14.5, but expression level declined (arrow). **C**) Immunofluorescence staining of Smad1, Smad5 and Smad8 (bottom panel). Negative control staining sections were incubated without primary antibodies (top bottom panel). At E12.5, Smad1, Smad5 and Smad8 were all localized in the bladder epithelium and body wall and urethra. At E14.5 Smad1 was localized in the transitional epithelium and muscularis mesenchyme and less intensely in the detrusor muscle (arrow). *Insert: positive staining of phosphorylated Smad1*. At E14.5, Smad5 and Smad8 showed similar expression pattern to Smad1. Both Smads were localized in the transitional epithelium and expressed in the muscularis mesenchyme (arrow). *Insert: positive staining of phosphorylated Smad5 and Smad8*. At E16.5, Smad1 and Smad5 showed strong expression in the transitional epithelium and lamina propia and sporadic expression in muscularis mesenchyme, but Smad8 ocalized less intensely in the transitional epithelium and muscularis mucosa (arrow). Magnification ×40. e: epithelum, l: lumen, bw: body wall, u: urethra, lp: lamina propia, mm: muscularis mesenchyme, dm: detrusor muscle. (Blue color: DAPI, Green color: Respective Smads).

BMP-4 mediates inductive interactions at several stages of urogenital development [20], [21], [22], [23]. BMP-4 activation is mediated by receptor-regulated Smads, Smad1, Smad5 and Smad8. To investigate their roles in BMP-4-dependent bladder development, we examined their spatial expression. Figure 3A (top panel) shows the H & E staining of bladder anatomy from E12.5 to E16.5. *In situ* hybridization of BMP-4 and immunofluorescence of Smad1, Smad5 and Smad8 results showed that, at the very early stage of bladder development (E12.5), BMP-4, Smad1, Smad5 and Smad8 were all detected in the bladder urothelium and urethra (Figs. 3B middle panel and 3C bottom panel). As bladder development continued to E14.5, BMP-4 was localized adjacent to the epithelium, lamina propia and muscularis mesenchyme and in the periphery of muscularis mucosa (Fig. 3B middle panel). Similar to BMP-4 expression, at E14.5, Smad1 was localized in the transitional epithelium and surrounding area of the lamina propia and muscularis mesenchyme (Fig. 3C bottom panel). At this stage, phosphorylated Smad1 was detected within the nuclei (Fig. 3C insert) of transitional epithelial cells, suggesting transcriptional activation of Smad1 in early bladder development and subsequent smooth muscle cell differentiation. As bladder development progressed to E16.5, BMP-4 showed a similar expression pattern as E14.5, but the expression was less intense at E16.5 (Fig. 3B). On the other hand, at E16.5, Smad1 was localized in the transitional epithelium and muscularis mesenchyme (Fig. 3C), suggesting that Smad1 might play a critical role in the epithelial-mesenchymal interaction for the differentiation of bladder smooth muscle cells. These results indicate a significant functional role of BMP-4 signaling during continued development of the bladder epithelium and ultimately for formation of the bladder smooth muscle layer. It is apparent that, at E16.5, the bladder epithelium and lamina propia showed strong Smad1 expression (Fig. 3C), indicating a functional role for BMP-4 signaling in this region of the epithelium. In the early smooth muscle differentiation stage (E14.5), Smad1 became localized in the transitional epithelium, but at the late smooth muscle differentiation (E16.5), Smad1 was localized in the transitional epithelium and lamina propia and the region between the muscularis mesenchyme and detrusor muscle (Fig. 3C), indicating an active functional role of BMP-4 during ongoing smooth muscle differentiation. No staining was observed when primary antibody was omitted (Fig. 3B BMP-4 negative control and Fig. 3C Smad1, -5 and -8 negative control).

BMP-responsive Smads, Smad5 and Smad8 showed comparatively similar expression patterns to that of Smad1. At E12.5, Smad5 and Smad8 were localized in the bladder epithelium and urethra (Fig. 3C bottom panel). With continuing bladder development to E14.5, Smad5 and Smad8 were localized in the transitional epithelium, lamina propia and muscularis mesenchyme. Compared to Smad5, Smad8 showed less intense expression in the muscularis mesenchyme, while the detrusor muscle area did not express Smad5 and Smad8 (Figs. 3C). At E16.5, when bladder smooth muscle differentiation occurs, Smad5 and Smad8 were localized in the transitional epithelium, lamina propia and muscularis mesenchyme (Fig. 3C). No staining was observed when primary antibody was omitted (Fig. 3C). These results suggest a further fine-tuning of the Smad mediated regulatory process.

### Expression pattern of TGF-β1 and TGF-β responsive Smads: Smad2 and Smad3

**Figure 4:**
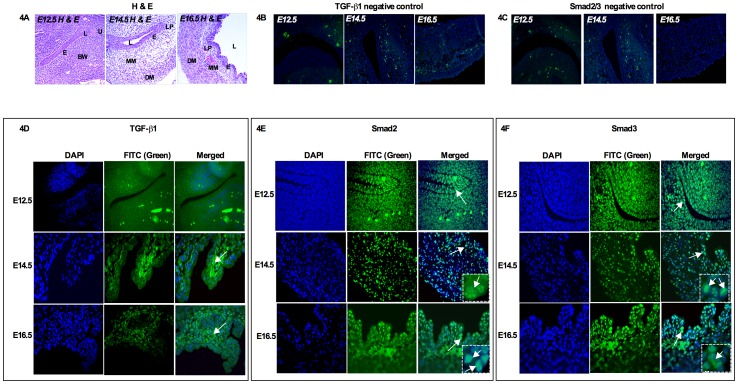
Expression pattern of TGF-β1 and TGF-β responsive Smads, Smad2 and Smad3. **A**) Top left panel shows H & E staining of mouse bladder anatomy from E12.5 to E16.5. **B**) Negative control sections of TGF-β1 and **C**) Smad2 and Smad3 incubated only with secondary antibodies (no primary antibodies, top middle and right panel). **D**) At E12.5, TGF-β1 expression was faint and localized in the epithelium and urethra (bottom left panel). At E14.5 and E16.5, TGF-β1 was localized in the transitional epithelium, adjacent to the lamina propia and muscularis mesenchyme (arrow). **E and F**) At E12.5, Smad2 and Smad3 were in the bladder epithelium and urethra (arrow, bottom middle and right panel). At E14.5, Smad2 and Smad3 were localized in the transitional epithelium, lamina propia and muscularis mucosa (arrow). *Insert: positive staining of phosphorylated Smad2 and Smad3* (bottom middle right panel). At E16.5, Smad2 was localized in the transitional epithelium and periphery of the detrusor muscle (arrow, bottom middle panel) and Smad3 was localized in the transitional epithelium, muscularis mesenchyme and periphery of the detrusor muscle (arrow, bottom right panel). *Insert: positive staining of phosphorylated Smad2 and Smad3*. Magnification ×40. e: epithelum, l: lumen, bw: body wall, u: urethra, lp:- lamina propia, mm: muscularis mesenchyme, dm: detrusor muscle. (Green color: Respective Smads, Blue color: DAPI).

TGF-β1 plays a key role during bladder development. TGF-β1 is expressed in the peripheral mesenchyme of the developing bladder [17]. Furthermore, Smad2 and Smad3 are intracellular mediators of TGF-β signaling [23]. To understand the biological significance of Smad2 and Smad3 in TGF-β-dependent bladder development, we examined their spatial localization. Figure 4A shows the H & E staining of bladder anatomy from E12.5 to E16.5. No staining was observed when primary antibody was omitted (Fig. 4B TGF-β1 and Fig. 4C Smad2 and Smad3). At the earliest stage of bladder development (E12.5), TGF-β1 showed faint expression in the bladder epithelium compared to urethra (Fig. 4D bottom left panel). As bladder development progressed to E14.5, TGF-β1 was found to be expressed in the transitional epithelium and muscularis mesenchyme and the region between the muscularis mesenchyme and detrusor muscle area (Fig. 4D). At E16.5, TGF-β1 expression became restricted to the transitional epithelium and muscularis mesenchyme (Fig. 4D).

We further investigated TGF-β responsive R-Smads, Smad2 and Smad3. At the early stage of bladder development (E12.5), Smad2 was localized in the epithelium and urethra (Fig. 4E bottom middle panel). As bladder development continued to earliest smooth muscle differentiation stage (E14.5), the transitional epithelium and muscularis mesenchyme showed positive staining of Smad2 (Fig. 4E) and, at E16.5, Smad2 expression was stronger in the transitional epithelium and in the periphery of the muscularis mesenchyme (Fig. 4E), suggesting the functional role of TGF-β in regulating bladder development and smooth muscle differentiation.

In contrast to Smad2, Smad3 expression showed continued expression in the transitional epithelium, muscularis mesenchyme and peripheral muscularis mesenchyme throughout bladder development. At E12.5, Smad3 was localized in the bladder epithelium and urethra, (Fig. 4F bottom right panel) and, as bladder morphogenesis continued to E14.5, Smad3 was localized in the transitional epithelium, lamina propia and muscularis mesenchyme (Fig. 4F). At the smooth muscle differentiation stage (E16.5), Smad3 expression was localized in the transitional epithelium and muscularis mesenchyme and in the periphery of the muscularis mesenchyme (Fig. 4F), which indicates a possible role of epithelial-mesenchymal interaction during bladder development. Furthermore, Smad3 nuclear expression at E14.5 and E16.5 was identical, which further underscores the functional role of Smad3 in TGF-β mediated bladder development and smooth muscle differentiation.

### Expression pattern of co-Smad, Smad4

**Figure 5:**
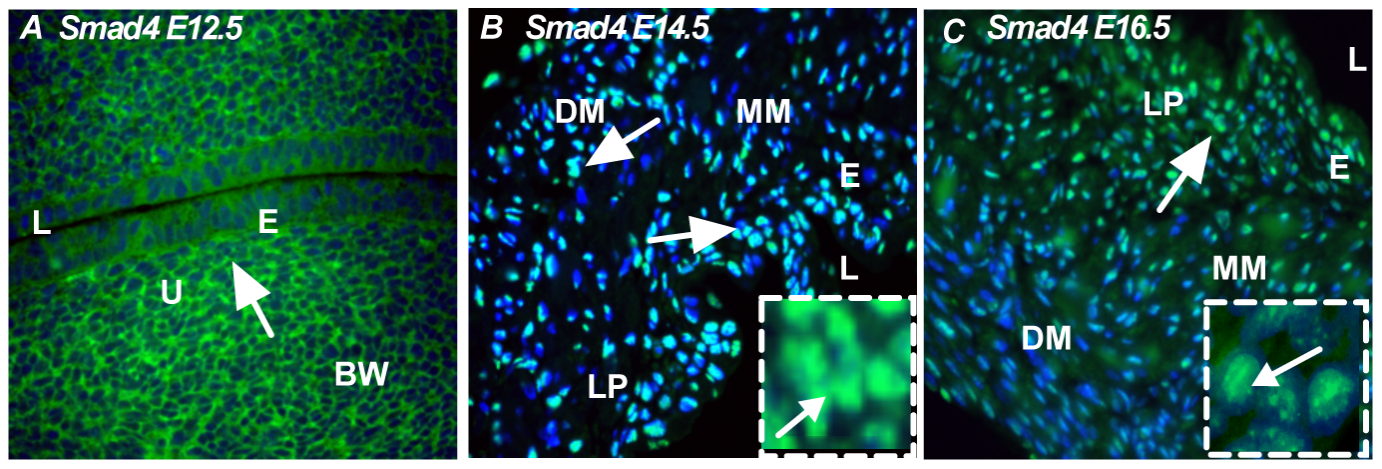
Expression pattern of common Smad, Smad4. **A**) At E12.5, Smad4 was localized in the bladder epithelium and urethra (arrow). **B**) At E14.5, Smad4 was localized in the transitional epithelium, muscularis mesenchyme, lamina propia and detrusor muscle (arrow) *Insert: nuclear localization of Smad4*. **C**) At E16.5, Smad4 was localized in the inner epithelium, less intensely in the muscularis mesenchyme and detrusor muscle (arrow) *Insert: nuclear localization of Smad4*. Magnification ×40. e: epithelum, l: lumen, bw: body wall, u: urethra, lp: lamina propia, mm: muscularis mesenchyme, dm: detrusor muscle. (Green color: Smad4, Blue color: DAPI).

Smad4 forms a complex with receptor-specific Smad proteins and translocates to the nucleus upon activation of the signaling pathway [24], [25]. Smad4 forms a complex with Smad1, Smad5 and Smad8 when BMP signaling is activated, whereas it complexes with Smad2 and Smad3 upon activation of the TGF-β or activin pathways. Accordingly, we found a moderate level of Smad4 expression in the bladder epithelium and urethra at the early stage (E12.5) of bladder development (Fig. 5A). However, at E14.5, Smad4 nuclear expression was strong in the transitional epithelium, lamina propia, muscularis mesenchyme and detrusor muscle (Fig. 5B, insert-nuclear localization of Smad4). At E16.5, Smad4 expression was similar to that of E14.5 (Figs. 5B and 5C, insert-nuclear localization of Smad4), suggesting continued association of TGF-β and BMP-4 signaling in regulating bladder development.

### Expression pattern of I-Smads: Smad6 and Smad7

**Figure 6:**
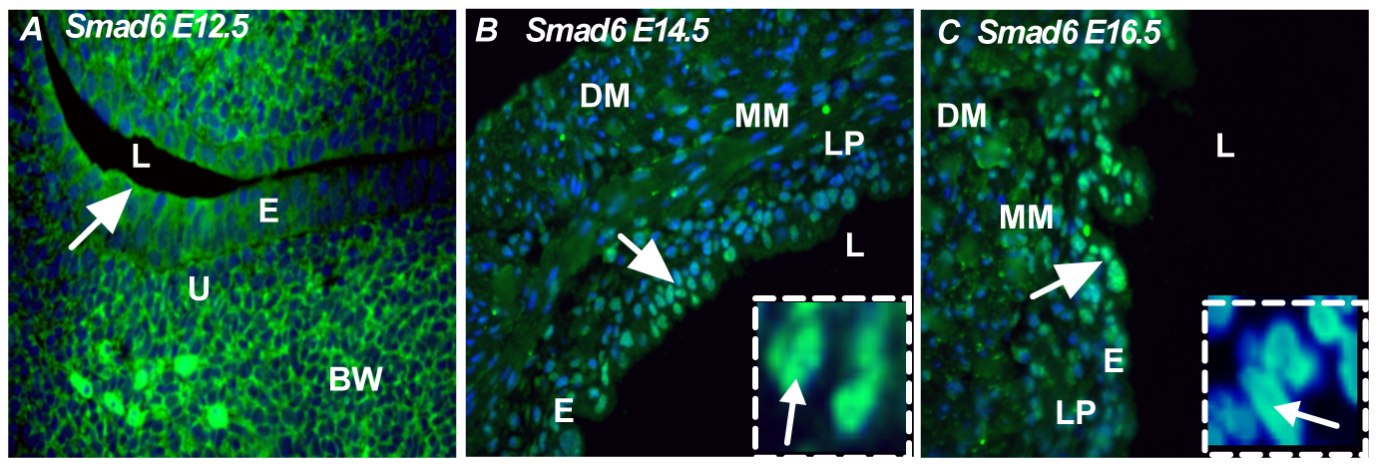
Expression pattern of inhibitory Smad, Smad6. **A**) At E12.5, Smad6 was localized in the bladder epithelium (arrow). **B**) At E14.5, Smad6 was localized in the transitional epithelium (arrow). **C**) At E16.5, Smad6 was predominantly localized in the transitional epithelium and less intensely in the muscularis mesenchyme (arrow) *Insert: nuclear localization of Smad6*. Magnification ×40. e: epithelum, l: lumen, bw: body wall, u: urethra, lp: lamina propia, mm: muscularis mesenchyme, dm: detrusor muscle. (Green color: Smad6, Blue color: DAPI).

**Figure 7:**
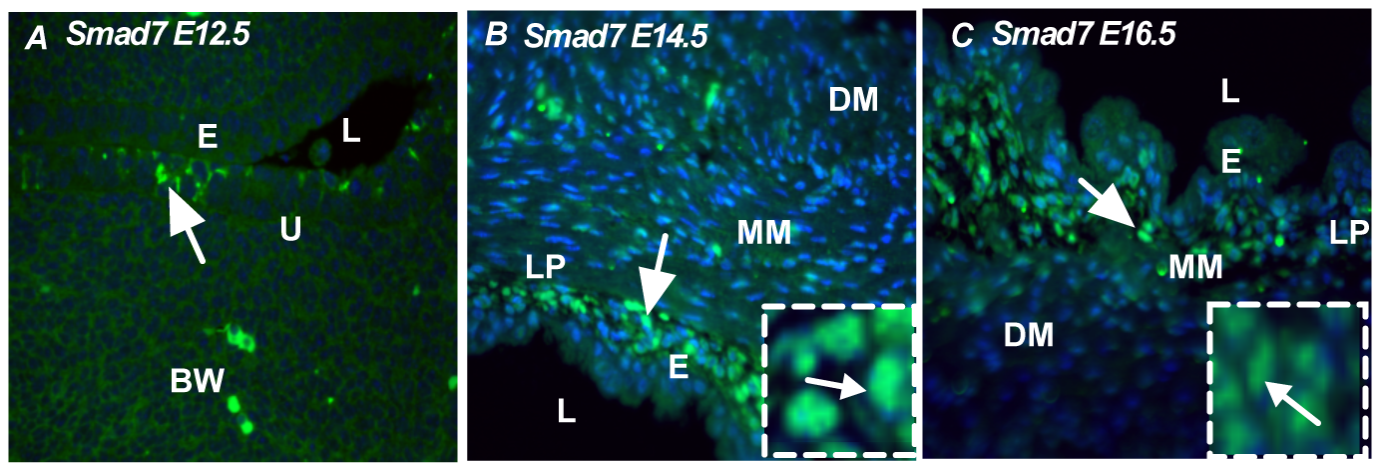
Expression pattern of inhibitory Smad, Smad7. **A**) At E12.5, Smad7 was less intense and localized in the bladder epithelium (arrow). **B**) At E14.5, Smad7 was localized in the transitional epithelium, lamina propia (arrow) *Insert: Nuclear localization of Smad7*. **C**) At E16.5, Smad7 was predominantly localized in lamina propia and less intensely in the muscularis mucosa (arrow) *Insert: nuclear localization of Smad7*. Magnification ×40. e: epithelum, l: lumen, bw: body wall, u: urethra, lp: lamina propia, mm: Muscularis mesenchyme, dm: detrusor muscle. (Green color: Smad7, Blue color: DAPI).

The role of inhibitory Smads is to inhibit the signaling activity of receptor-regulated Smads. It has been shown that Smad6 inhibits BMP signaling [26], [27], whereas Smad7 preferentially inhibits TGF-β signaling. Interestingly, we found that nuclear localization of both Smad6 and Smad7 was detected throughout bladder development. At the early stage of bladder development (E12.5), Smad6 and Smad7 were localized in the bladder epithelium (Figs. 6A and 7A). At the initiation of the smooth muscle differentiation stage (E14.5), Smad6 and Smad7 were restricted to the transitional epithelium and muscularis mesenchyme (Figs. 6B and 7B, insert-nuclear localization of Smad6 and Smad7) and, at E16.5, Smad6 was restricted to the transitional epithelium and not detected in the peripheral muscularis mesenchyme and detrusor muscle (Fig. 6C). At E16.5, Smad7 expression was restricted in the bladder epithelium and muscularis mesenchyme (Fig. 7C). These results indicate that Smad6 and Smad7 were present to exert an inhibitory control on TGF-β and BMP-4 mediated signaling throughout bladder development and early smooth muscle cells formation.

### In vitro bladder organ culture

**Figure 8:**
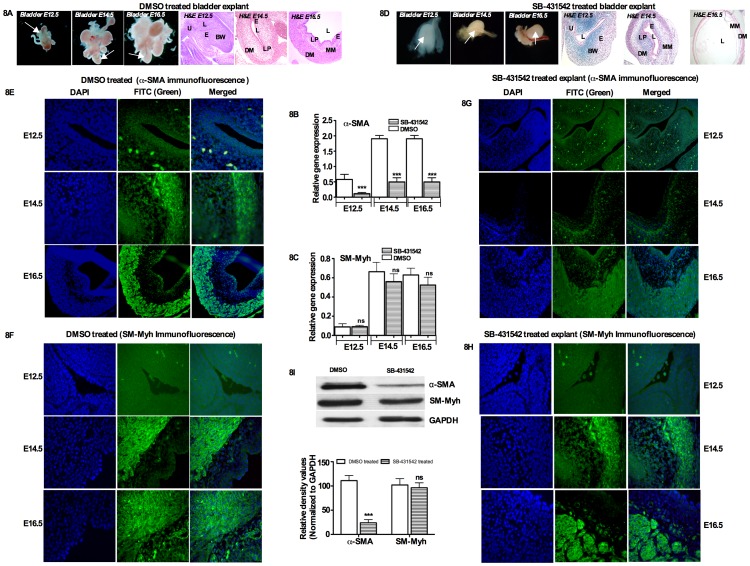
Effects of SB-431542 in bladder smooth muscle cell differentiation: A) Top left panel. E12.5, E14.5 and E16.5 bladders were cultured in DMEM/F12 50∶50 supplemented with insulin-transferrin and penicillin/streptomycin antibiotics. Bladder explants cultures treated for three days in DMSO only and showing H & E staining sections. (Phase contrast microscope Magnification 4×). **D) Top right panel:** Bladder explants culture treated in TβRI inhibitor SB-431542 for three days and showing with H & E staining section. (Phase contrast microscope Magnification 4×). **B and C**) α-SMA and SM-Myh mRNA gene expression against GAPDH by qRT-PCR at E12.5 to E16.5 in cultured embryonic bladders after three days of organ culture (middle panel). **E**) Immunofluorescence staining of α-SMA and SM-Myh and expressed at embryonic days from E14.5 to E16.5 in the untreated bladder explants (middle top panel) and α-SMA expression was faint at E14.5 to E16.5 after three days treatment with SB-431542 (top right panel). **F and H**) In contrast, SM-Myh expression showed no changes in the untreated and treated group (bottom left and right panel). **I**) Western blot analysis showing the expression of α-SMA and SM-Myh with densitometry analysis. In SB-431542 treated bladder explants, α-SMA was decreased by 90%, but no significant changes were observed in SM-Myh. e: epithelium, l: lumen, bw: body wall, u: urethra, lp: lamina propia, mm: muscularis mesenchyme, dm: detrusor muscle. (Green color: α-SMA and SM-Myh, Blue color: DAPI).

We next asked if inhibition of TGF-β signaling would disrupt bladder development starting from the earliest stage (E12.5). Figures 8A and 8D (top left and right panel) show the phase contrast microscopy and H & E staining of mock (DMSO) and TβRI inhibitor SB-431542 treated cultured bladders. No significant changes were observed between the treated and untreated bladder explants group. When E12.5, E14.5 and E16.5 bladders were cultured without inhibitor (medium and DMSO only), α-SMA and smooth muscle myosin heavy chain (SM-Myh) mRNA induction was detected after three days of bladder organ culture in all embryonic cultured bladders (Figs. 8B, 8C middle panel). Bladders treated with SB-431542 showed a significant decrease in α-SMA expression in all embryonic stages in mRNA levels (α-SMA: E12.5 *p = 0.004*, E14.5 *p = 0.0001*, E16.5 *p = 0.001*), but no significant changes were observed for SM-Myh mRNA expression (SM-Myh: E12.5 *p = 0.919*, E14.5 *p = 0.6236* and E16.5 *p = 0.0130*) (Figs. 8B, 8C). Immunofluorescence staining revealed that α-SMA and SM-Myh expression was faint in the outer layer of the urothelium after three days of E12.5 bladder culture (Fig. 8E left panel). Compared to E12.5, E14.5 and E16.5 cultured bladders showed strong expression of α-SMA and SM-Myh after three days of culture (Figs. 8E, 8F left panel). TβRI inhibitor SB-431542 treated cultured bladders showed faint expression of α-SMA at E14.5 and E16 (Fig. 8G right panel), corroborating the mRNA and Western blot results, but no significant changes in the expression of SM-Myh at E14.5 and E16.5 (Fig. 8H). We further confirmed our results by Western blot analysis and showed that, compared to DMSO treated bladder explants, α-SMA protein expression was decreased by 90% when the explants were treated with TβRI inhibitor SB-431542, while SM-Myh expression did not show any significant change in protein levels (Fig. 8I).

**Figure 9:**
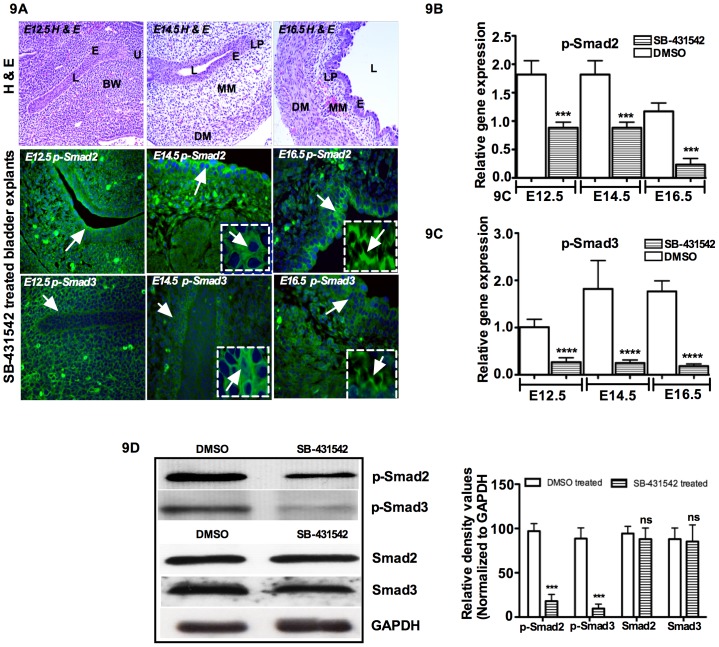
Induction of α-SMA by TGF-β in cultured bladder organ requires Smad2 and Smad3. E12.5, E14.5 and E16.5 bladders were cultured in DMEM/F12 50∶50 supplemented with insulin-transferrin and penicillin/streptomycin antibiotics. **A) Top panel:** H & E staining of bladder anatomy from E12.5 to E16.5. Blockade of Smad2 and Smad3 by SB-431542 in cultured bladder as detected by immunofluorescence. Cultured bladders were treated with SB-431542 for three days and processed for immunofluorescence using p-Smad2 and p-Smad3 antibodies. *Insert: staining of phosphorylated Smad2 and Smad3*. Nuclei of the same cells stained with DAPI. **B and C)** p-Smad2 and p-Smad3 mRNA gene expression against GAPDH by qRT-PCR at E12.5 to E16.5 in cultured embryonic bladders after three days with and without SB-431542. **D**) Western blot analysis showing the expression of p-Smad2 and p-Smad3 with densitometry analysis. In SB-431542 treated bladder explants, p-Smad2 was decreased by 85% and p-Smad3 was decreased by more than 95%. No changes were observed for total Smad2 and Smad3 expression. Magnification ×40. e: epithelum, l: lumen, bw: body wall, u: urethra, lp: lamina propia, mm: muscularis mesenchyme, dm: Detrusor muscle. (Green color: Respective Smads, Blue color: DAPI).

To determine whether α-SMA and SM-Myh expression was Smad2 and Smad3 dependent, we used TβRI inhibitor SB-431542 to block phosphorylation of Smad2/3 and Smad4 complex formation downstream from TGF-β signaling and smooth muscle formation, and we analyzed p-Smad2/3 expression in SB-431542 treated organ cultures. Figure 9A (top panel) shows the H & E staining of bladder anatomy. In SB-431542 treated cultures of E12.5 bladders, qRT-PCR data showed that p-Smad2 (*p = 0.0058*) and p-Smad3 (*p = 0.002*) gene expressions were significantly decreased compared to untreated bladders, which was confirmed by Western blot analysis (Figs. 9B, 9C and 9D). Similar to E12.5, E14.5 and E16.5 showed decreased gene expression of p-Smad2 (E14.5- *p = 0.001*, E16.5- *p = 0.008*) and Smad3 (E14.5- *p = 0.0001*, E16.5- *p = 0.001*) (Figs. 9A, 9B, 9D). Our immunofluorescence results showed that phosphorylation of Smad2 and Smad3 was prevented by SB-431542 treatment from E12.5 to E16.5 (Fig. 9C). Western blot results for p-Smad2 and p-Smad3 revealed that, in bladder explants treated with TβRI inhibitor, p-Smad2 protein expression decreased by 85%, whereas p-Smad3 expression decreased by 95% compared to DMSO treated bladder explants. No changes were observed in total Smad2 and Smad3 expression (Fig. 9D). These results demonstrate that inhibiting TGF-β signaling with SB-431542 blocks phosphorylation of Smad2 and Smad3 and prevents nuclear translocation of p-Smad2 and p-Smad3, which ultimately interrupts bladder smooth muscle formation. This *in vivo* data identify TGF-β responsive Smad2 and Smad3 as important developmental regulators in bladder development and early smooth muscle cell formation.

## Discussion

**Figure 10:**
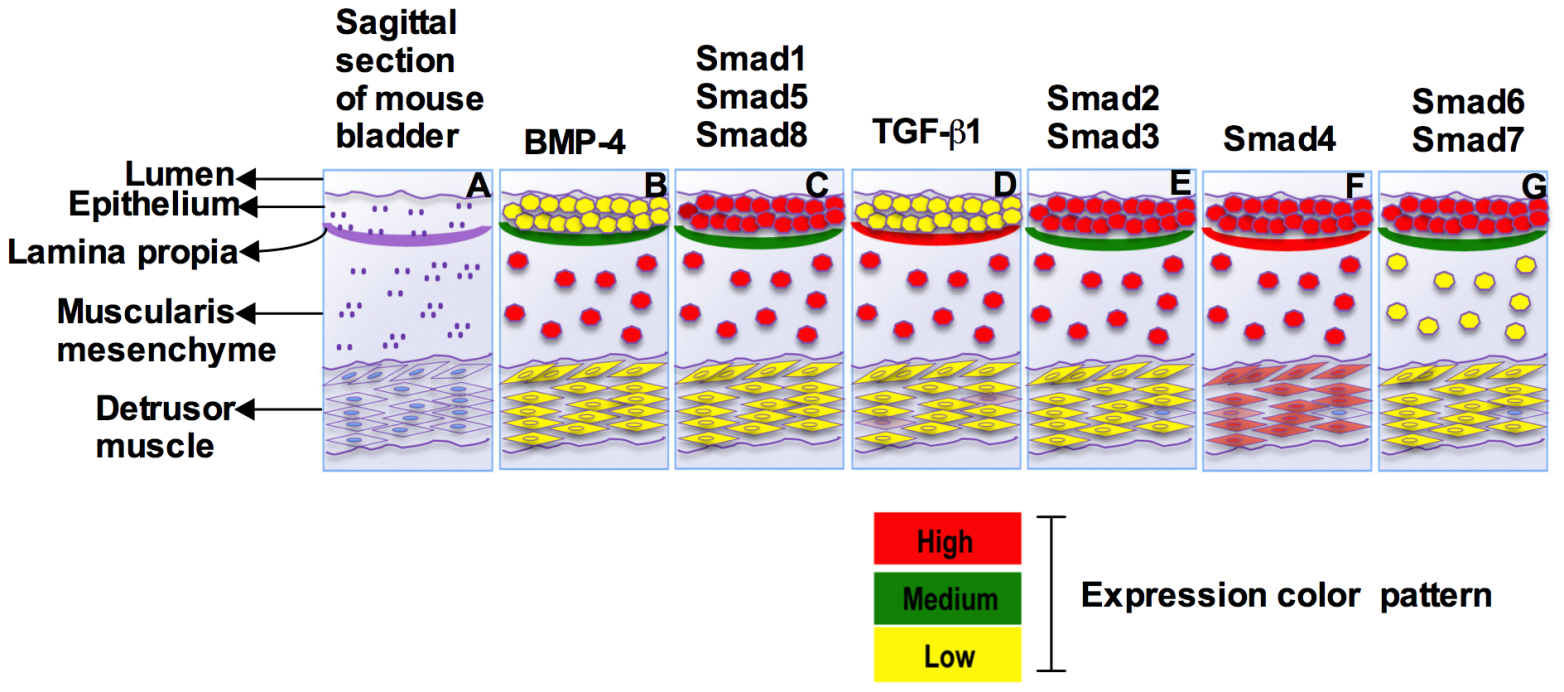
Expression pattern summary of Smads. ISH and Immunofluorescence staining revealed expression pattern of BMP-4, TGF-β1 and Smads based on intensity of expression for particular Smads within the bladder cellular compartment at E14.5 and six different kinds of expression pattern summarized below: **A**) Sagittal section of mouse bladder. **B**) BMP-4 was strongly expressed in the muscularis mesenchyme, medium in the lamina propia and weaker in the epithelium and detrusor muscle. **C**) Smad1, Smad5 and Smad8 were highly expressed in the bladder epithelium and muscularis mesenchyme, medium level in the lamina propia and weaker expression in the detrusor muscle. **D**) TGF-β1 highly expressed in the lamina propia and muscularis mesenchyme and weaker expression in epithelium and detrusor muscle. **E**) Smad2 and Smad3 were highly expressed in the epithelium and muscularis mesenchyme, medium level in the lamina propia and weaker expression in the detrusor muscle. **F**) Smad4 expression level was high in the epithelium, lamina propia, muscularis mesenchyme and detrusor muscle respectively and **G**) Smad6 and Smad7 were highly expressed in the epithelium but medium level in the lamina propia and weaker expression in the muscularis mesenchyme and detrusor muscle.

The urinary bladder develops from endoderm-derived epithelial cells as well as mesenchymal cells stemming from the urogenital sinus and allantois. In CD1 mice, the developing bladder is first morphologically distinguishable at E12.5 [28]. During bladder development, a gradual transition occurs from an undifferentiated mesenchyme into a differentiated smooth muscle layer [29], [30]. The differentiation of mesenchyme to smooth muscle is brought about by diffusible growth factors secreted by the epithelium [17]. Despite the reports about the roles of TGF-β and BMP-4 in bladder development, we were unaware of any studies documenting the expression pattern of Smads in the developing bladder. Hence, in the present study, we examined the cell type specific temporal and spatial expression pattern of Smads during mesenchymal differentiation of embryonic bladder and the critical roles of TGF-β responsive R-Smads, Smad2 and Smad3 in bladder smooth muscle differentiation. The results presented here highlight an important role for TGF-β1 and differential expression of R-Smads, co-Smad and I-Smads during mouse bladder development, with each Smad showing particular expression patterns during bladder development (Fig. 10).

BMP-4 expression has been observed in the embryonic bladder [28]. BMP-4 is essential for mesoderm formation [31] and is also involved in epithelial to mesenchymal interaction during embryogenesis [32], urogenital development [12] and smooth muscle differentiation in the ureter [33]. We found that, similar to TGF-β1, BMP-4 is expressed in the muscularis mesenchyme and lamina propia at E14.5. This raises the possibility that both BMP-4 and TGF-β collectively play a role in regulating smooth muscle formation and differentiation as both pathways participate in cell proliferation, differentiation, apoptosis and migration [34], [35]. Smad1 knockout mice exhibit embryonic lethality prior to E10.5 due to extra embryonic defects [36], [37]. Smad5 knockout mice show embryonic lethality and allantois shortening, defects in cardiac development and craniofacial development [38]. These findings demonstrate that both of these BMP responsive Smads are essential in early embryogenesis. In our study, BMP-4 expression was observed in the mesenchymal layer, in agreement with a previous study in which BMP-4 expression was confined to the mesenchyme [19], [39]. On the other hand, though the BMP responsive Smad1 and Smad5 were localized in the bladder epithelium/urothelium, Smad8 was exclusively expressed in the muscularis mesenchyme. Hence, we note that the spatial and temporal expression pattern of Smad8 is similar to BMP-4, suggesting that Smad8 is likely a major mediator within BMP responsive cells.

On the other hand, the presence of Smad1 and Smad5 in bladder epithelium/urothelium suggests that BMP-4 expressed in mesenchyme may have some activity in the maintenance of epithelium/urothelium. Previous studies have focused on the role of epithelium in differentiation of mesenchyme. However, the influence of mesenchyme on maintaining the phenotype of bladder urothelium is also a possibility, and may be influenced by BMP-4. Nevertheless, the role of BMP-4, expressed in the mesenchymal compartment, in the restoration of urothelium after uropathogenic infection has been reported [40]. Hence, we speculate that Smad1 and Smad5 may have a role in mediating the maintenance of bladder epithelium/urothelium via BMP-4 signaling.

The TGF-β superfamily plays a significant critical role during normal development of the urogenital system [41]. TGF-β1 has been shown to stimulate cell growth and up-regulation of smooth muscle cell differentiation *in vitro* [42]. In our study, we found that TGF-β1 was strongly expressed in the peripheral mesenchyme. This is in agreement with previous data, which showed highest expression of TGF-β1 at E13.5 in the developing bladder [17]. In addition, TGF-β1 was highly expressed in the epithelium/urothelium and lamina propria. TGF-β responsive Smad2 and Smad3 were also localized in the nuclei of both epithelial and mesenchyme cells. This supports the active involvement of phosphorylated Smad2 and Smad3 in TGF-β mediated smooth muscle differentiation during early bladder development. In the muscularis mesenchyme, Smad3 was strongly expressed whereas Smad2 expression was low or faint. Their activity in the muscularis mucosa has to be considered in context with Smad4, since Smad4 is required for the translocation of regulatory Smads into the nucleus and is the common mediator for Smad-dependent signaling for TGF-βs, BMPs and activins [34]. In a recent paper [18], *Gli2* was shown to mediate the inductive effect of Shh signaling on mesenchymal proliferation as well as the radial patterning of smooth muscle in the bladder, possibly through the regulation of BMP-4. Since TGF-β can induce the expression of *Gli1* and *Gli2* through a Smad3-dependent pathway [43] and since the spatial distribution of Smad2 and Smad3 matches with the distribution of the TGF-β ligand in early bladder development, it is possible that Smad2 and Smad3 might be involved in the cross talk of TGF-β, Shh and BMP-4 signaling pathways during smooth muscle differentiation.

All activated R-Smads translocate into the nucleus in a complex form with common Smad, Smad4 to regulate downstream gene transcription [24], [25]. Thus, Smad4 is at the core of the transcriptional responses in the TGF-β and BMP-signaling pathway. In our study, we found that Smad4 was localized in the bladder epithelium, muscularis mesenchyme and detrusor muscle. This expression pattern suggests that Smad4 functions as a critical mediator for transducing signaling initiated by TGF-β and BMP. In our study, we provided supportive data for the possible involvement of TGF-β, BMP-4 and Smads signaling, and their possible interaction among these intracellular signaling mediators. Thus Smad4 plays a strategically critical role during bladder development by mediating signaling from TGF-β and BMPs.

The inhibitory Smads inhibit TGF-β and BMP signaling by preventing phosphorylation of pathway specific Smads. Smad6 and Smad7 has been shown to inhibit signaling downstream of BMP receptors and prevent formation of complex of Smad4-R-Smads [44]. Thus, Smad7 acts as a general TGF-β family inhibitor and Smad6 preferentially inhibits BMP signaling. Taken together in our study, the inhibitory Smads, Smad6 and Smad7 were found to have been expressed throughout bladder development, suggesting the possibilities that Smad7 may inhibit growth arrest and apoptosis, while Smad6 participates in a negative feedback loop to control growth arrest and apoptosis. Thus, our observation suggests that the inhibitory Smads may function to fine tune the spatial and temporal functioning of transcriptionally active Smads.

The bladder is a hollow smooth muscle organ, covered on its outer aspect by serosa and fascia. The bladder muscular wall is formed of smooth muscle cells, which comprise the detrusor muscle. During filling of the urinary bladder, the smooth muscle cells have to relax and this process is regulated by signaling pathways. Functional changes of the bladder can be found in several clinically important conditions such as lower urinary tract symptom (LUTS) and bladder outlet obstruction [45]. There are four different isoforms of actin expressed in smooth muscle, α, β and two isoforms of γ-actin [46]. The most prominent isoforms in rat and human are α- and γ-actin. α-smooth muscle actin (α-SMA) is commonly used as a marker of smooth muscle cells as it is predominantly found in smooth muscle, but it may also be located in fibroblasts and myofibroblasts, the vascular network, the airway and the liver. SMCs are defined by specific molecular markers and contractile functions. α-SMA is the early marker of developing SMCs while SM-Myh is the late marker. The principal function of bladder SMC is to regulate pressure during filling and contractility during emptying [47]. Smads are major intracellular mediators of TGF-β signaling and, after phosphorylation of Smad2 and Smad3, they translocate to the nucleus to regulate gene transcription. Embryonic bladders grown in organ culture were used to test our hypothesis that TGF-β responsive intracellular signaling molecules Smad2 and Smad3 mediate bladder smooth muscle formation. Our results demonstrate that α-SMA, Smad2 and Smad3 expression remains relatively similar in mRNA and protein levels. But disruption of complex formation between Smad2/3 and Smad4 by TGF-β inhibitor SB-431542 significantly decreases expressions of these genes (α-SMA, Smad2 and Smad3) as evidenced by qRT-PCR, immunofluorescence and Western blot. Based on this *in vitro* demonstration that α-SMA expression was blocked by TβR1 inhibitor SB-431542 correlating to the absence of Smad2 and Smad3 expression, suggests that signaling through this pathway is required for early stages of bladder development and multi-stage transition of bladder urothelial cells to mesenchymal cells phenotype. Our results demonstrate that cultured bladders treated with TGF-β1 and TGF-β1 inhibitor SB-431542 do not express α-SMA, but, surprisingly, SM-Myh was still expressed. The molecular mechanism that mediates the differentiation of SM-Myhduring bladder development is not well studied; however, Baskin et al. [48] postulated that TGF-β2 and TGF-β3 might regulate the expression of SM-Myh during SMC differentiation. In addition to TGF-β, Notch signaling induces the SMC differentiation during development in many cell types and tissues [49], [50]. It appears that TGF-β may cross talk with other signaling pathways in the regulation of SMC differentiation. Taken together, these data support our hypothesis that TGF-β/Smad2/3 signaling is required at the early stage in the process of bladder development prior to induction of α-SMA. We assume, therefore, that Smad2 and Smad3 may be mediating early bladder development, and further studies are needed to investigate the potential functional roles of Smad2 and Smad3 in regulating TGF-β signaling during bladder development.

In summary, the differential localization of Smads in mouse embryonic bladder provides new insight into the involvement of TGF-β1 and BMP-4 in bladder development. Expression of the different Smads was found to be both widespread and selective during bladder organogenesis, suggesting that each Smad may have a distinct influence on the development of cellular phenotypes and organogenesis. Although the complete sequence of molecular events involved in Smad2/3-dependent bladder development is still to be clarified, we have been able to characterize some of the early events in the process. We therefore suggest that further investigation of signaling cross talk between Smad-dependent and Smad-independent pathways may be necessary to fully understand the complex signaling cascade and mechanism during bladder development. Manipulation of individual Smads by ectopic expression and knockout analysis could provide information about the roles of individual Smads and their requirements for early bladder development.

## Materials and Methods

### Embryos and tissue collection

In our studies, we used normal, timed mated outbreed CD1 mice (Charles River Valley, QC). Bladders were microdissected at E12.5, E14.5, E16.5, E18.5 and neonatal Day0 days (n = 3). For *in situ* hybridization and immunostaining studies, whole embryos were fixed in 4% paraformaldehyde in PBS and, for mRNA expression analysis, embryonic bladders were microdissected and snap frozen in liquid nitrogen. Total RNA was isolated (10 embryonic bladders/stage) using the RNeasy Mini Kits (Qiagen, Valencia, CA). All procedures were performed in ice cold PBS. All animal experiments were performed with the approval of the laboratory animal services (LAS) at the Hospital for Sick Children, Toronto, Canada.

### In vivo RNA extraction and mRNA expression by qRT-PCR

The expression of BMP-4, TGF-β1, and Smad1, -2, -3, -4, -5, -6, -7, -8, mRNA was quantified by quantitative Real Time-Polymerase Chain Reaction (qRT-PCR). Total embryonic bladder RNA was extracted using RNeasy Mini Kit (Qiagen). Extracted total RNA was reverse transcribed into cDNA using Superscript II RT (Invitrogen, CA). Table 1 lists the primers sequences used. Relative mRNA expression was measured quantitatively using florescent SYBR Green qRT-PCR assay kit (Finnzymes, Finland) on a Chromo™ 4 Cycler. Glyceraldehyde 3-phosphate dehydrogenase (GAPDH) was used as an internal control for sample normalization. The PCR cycle conditions were as follows: 95°C for 15 minutes, 94°C for 60 s, 60°C for 45 s and 72°C for 60 s, and each sample was repeated in triplicate. Relative mRNA levels were calculated using Ct values. Each experiment was repeated three times, and the mean value was calculated as an expression value for each stage of embryonic bladder development.

**Table 1:** List of primer sequences.

*Genes*	*Forward primers*	*Reverse primers*
Smad1	5′AAG GTG GGG AAA GTG AAA C 3′	5′CTG CTT GGA ACC AAA TGG GAA 3′
Smad2	5′CCC ACT CCA TTC CAG AAA AC 3′	5′GAG CCT GTG TCC ATA CTT TG 3′
Smad3	5′GTT GGA CGA GCT GGA GAA G 3′	5′GTA GTA GGA GAT GGA GCA C 3′
Smad4	5′GTA GTA GGA GAT GGA GCA C 3′	5-ATG CTT TAG TTC ATT CTT GTG 3′
Smad5	5′GGA ACC TGA GCC ACA ATG AA 3′	5′CTT GCT GGG GAG TTG GGA TA 3′
Smad6	5′CCA CTG GAT CTG TCC GAT TC 3′	5′AAG TCG AAC ACC TTG ATG GAG 3′
Smad7	5′TCC TGC TGT GCA AAG TGT TC 3′	5′AGT AAG GAG GAG GGG GAG AC 3′
Smad8	5′CAC CGA CCC TTC CAA TAA C 3′	5′CTG GAC AAA GAT GCT GCT G 3′
BMP-4	5′CTC CCA AGA ATC ATG GAC TG 3′	5′AAA GCA GAG CTC TCA CTG GT 3′
TGF-β1	5′GTG CGG CAG CTG TAC ATT GAC TTT 3′	5′ TGT ACT GTG TGT CCA GGC TCC AAA 3′

### ISH on paraffin sections

Whole embryos were fixed in 4% paraformaldehyde in PBS for 16 h at 4°C. Slides were dewaxed and hydrated through a xylene-ethanol series 3×10 min xylene, 2×5 min 100%, 1×5 min 90%, 1×5 min 70%, 1×5 min 50% and 1×5 min 30% EtOH followed by 3×5 min in PBS washing. Sections were fixed in 4% paraformaldehyde in PBS for 20 min and washed with PBS for 5 min, permeabilzed for 30 min in proteinase K (20 mg/ml in PBS) at 37°C and washed in PBS 2×5 min each. Sections were treated with 0.2 N HCL to neutralize the samples (10 min) followed by washing with PBS (5 min). Sections were prehydrated with hybridization buffer (5×SSC, 50% formamide, yeast tRNA 50 µg/ml, Heparin 50 µg/ml and SDS 1%) and added 10 ng/ml RNA probe in the sections and placed the slides in a humidified plastic container and incubated overnight (18–20 hrs) at 55°C under the cover slip to prevent evaporation. Sections were rinsed with post hybridization buffers (5×SSC- preheated 60°C for 5 min, 50% formamide-preheated 60°C 15 min, TNE I-preheated 37°C 5 min, RNaseA:TNE-preheated 37°C 45 min, TNE II-preheated 37°C 5 min and 2×SSC at 55°C 15 min each). Sections were incubated with blocking solution (TBST and 10% blocking reagent) for 1 hr and incubated with α-DIG –AP (Roche 11093274910) at 1∶2000 in blocking buffer. Sections were rinsed with TBST-Lavamisole 3 times 10 min each and added BM Purple AP substrate to the embryo section (Roche 11442074001) and incubated over night for color development Finally the reaction was stopped by washing the slides in ddH_2_O several times and dehydrated through EtOH-xylene series and mounted xylene based Vecta Mount H-5000 for visualization. The BMP-4 probes were a kind gift from Dr. Norman Rosenblum, Hospital for Sick Children, Toronto, ON, Canada.

### Immunostaining

Tissue sections were deparaffinized in 100% xylene 3 times for 10 minutes each and hydrated in graded alcoholic solutions and washed with ddH_2_0. Antigen retrieval was carried out by microwave heating for 14 minutes in tri-sodium citrate buffer solution (pH 6.0). 4% BSA (Bovine serum albumin) was applied to tissue sections for 30 minutes to bind non-specific sites. The sections were then incubated with primary antibodies over-night at 4°C. The primary antibodies used were: anti-phospho-Smad1/5/8 (Cell Signaling, MA), anti-phospho-Smad2/3 (Santa Cruz, CA), anti-TβR1 (Santa Cruz, CA), anti-Smad6 and anti-Smad7 (Santa Cruz, CA), anti-Smad4 (Abcam, Cambridge, MA). After overnight incubation in primary antibodies sections were washed in PBS 3 times for 10 minutes each and incubated with secondary antibodies: donkey anti-goat and chicken anti-rabbit (Invitrogen, USA) diluted in 4% BSA for 1 hour at RT. For negative controls, sections were incubated without primary antibodies. Finally, sections were washed in PBS for 10 minute 3 times each and mounted with VectaShield mounting medium with DAPI.

### Western blot analysis

Total cell lysates of bladder tissues were obtained by incubating the tissues in RIPA buffer (Sigma, Canada) containing 50 mM Tris-HCL, 150 mM NaCl, 1% NP-40, 0.1 SDS, 0.5% sodim deoxycholate, 2 mM sodium fluoride, 2 mM Na_3_VO4_2_, 1 mM EDTA, 1 mM EGTA and supplemented with protease inhibitor cocktail (Roche). Protein concentrations were measured using BCA kits (Pierce) and 50 µg of protein/lane were separated using SDS-PAGE and transferred to nitrocellulose membrane (Bio-Rad). The membranes were blocked by blocking buffer containing 0.1% Tween 20 and 5% non-fat dry milk in PBS and incubated with primary antibodies. After incubation with HRP conjugated secondary antibodies immunodetection was performed using chemiluminescence (ECL, Amersham Life Science) and the signals were then detected using X-ray film. Equal loading of protein was confirmed by reprobing membranes for GAPDH. Experiments were repeated three times and one representative data set reported here.

### Embryonic bladder organ culture

Female pregnant CD1 mice were killed by cervical dislocation and the embryonic bladders from E12.5 to E16.5 removed aseptically. The isolated bladders were cultured on 1.0 µm pore cell culture insert (Cat no: 35-3103, Becton Dickinson, NJ) in DMEM/F12 50∶50 supplemented with insulin-transferrin and penicillin/streptomycin antibiotics. A total of ten (10) bladders from each embryonic stage were placed on each cell culture insert. Cultures were maintained for three days in a humidified atmosphere of 5% C0_2_ in air at 37°C and medium was renewed everyday. At the end of the three days embryonic bladders were harvested for histology and qRT-PCR analysis.

### Disruption of Smad2 and Smad3 pathway by SB-431542, a TGF-β receptor kinase inhibitor

We studied the role of TGF-β responsive R-Smads (Smad2 and Smad3) pathway in bladder smooth muscle differentiation *in vitro* by using small molecule inhibitor SB- 431542, a potent inhibitor of TβRI kinase activity. SB-431542 was added to culture medium at 10 µM. E12.5, E14.5 and E16.5 bladders were treated with SB-431542 for three days. SB-431542 was dissolved in DMSO at 10 µM and stored at −20°C. The untreated group of bladder explants was treated with an equal volume of DMSO.

### Statistical analysis

To determine the significance of differential expression patterns in the developing bladder, a one-sided Mann-Whitney U nonparametric analysis was performed, for which a *p*- value of *<0.05* was considered significant.
